# Pro-Inflammatory Chemokine CCL2 (MCP-1) Promotes Healing in Diabetic Wounds by Restoring the Macrophage Response

**DOI:** 10.1371/journal.pone.0091574

**Published:** 2014-03-11

**Authors:** Stephen Wood, Vijayakumar Jayaraman, Erica J. Huelsmann, Brian Bonish, Derick Burgad, Gayathri Sivaramakrishnan, Shanshan Qin, Luisa A. DiPietro, Andrew Zloza, Chunxiang Zhang, Sasha H. Shafikhani

**Affiliations:** 1 Department of Immunology/Microbiology, Rush University Medical Center, Chicago, Illinois, United States of America; 2 Rush University Cancer Center, Rush University Medical Center, Chicago, Illinois, United States of America; 3 Department of Dermatology, Rush University Medical Center, Chicago, Illinois, United States of America; 4 Developmental Center for AIDS Research, Rush University Medical Center, Chicago, Illinois, United States of America; 5 Department of Pharmacology, Rush University Medical Center, Chicago, Illinois, United States of America; 6 Center for Wound Healing and Tissue Regeneration, College of Dentistry, University of Illinois at Chicago, Chicago, Illinois, United States of America; Shanghai Medical College, China

## Abstract

Prior studies suggest that the impaired healing seen in diabetic wounds derives from a state of persistent hyper-inflammation characterized by harmful increases in inflammatory leukocytes including macrophages. However, such studies have focused on wounds at later time points (day 10 or older), and very little attention has been given to the dynamics of macrophage responses in diabetic wounds early after injury. Given the importance of macrophages for the process of healing, we studied the dynamics of macrophage response during early and late phases of healing in diabetic wounds. Here, we report that early after injury, the diabetic wound exhibits a significant delay in macrophage infiltration. The delay in the macrophage response in diabetic wounds results from reduced Chemokine (C-C motif) ligand 2 (CCL2) expression. Importantly, one-time treatment with chemoattractant CCL2 significantly stimulated healing in diabetic wounds by restoring the macrophage response. Our data demonstrate that, rather than a hyper-inflammatory state; the early diabetic wound exhibits a paradoxical and damaging decrease in essential macrophage response. Our studies suggest that the restoration of the proper kinetics of macrophage response may be able to jumpstart subsequent healing stages. CCL2 chemokine-based therapy may be an attractive strategy to promote healing in diabetic wounds.

## Introduction

Inflammation is a double-edge sword in wound healing. In normal settings, acute inflammatory response occurs early; it is necessary to fight off invading pathogens and to jumpstart healing through the production of chemokines, cytokines, and growth factors; but it also resolves quickly so as not to perturb subsequent stages of repair [1]. In pathological conditions such as diabetic ulcers, inflammation becomes locked in a non-resolving mode and is recognized as a major culprit that contributes to impaired healing in these settings [2], [3].

Macrophages are important components of the inflammatory response during wound healing as they destroy invading pathogens, clear cellular debris, and express a multitude of cytokines, chemokines, and growth factors, which are necessary to mediate subsequent healing stages [4]. Although, there are a few reports that suggest that specific types of wounds, including fetal wounds, can heal normally with minimal or in the absence of macrophages [5], [6], a preponderance of evidence points to a central role for macrophages in mediating effective repair in normal settings [7]–[11]. In the diabetic chronic wound setting however, prior studies suggest that the impaired healing derives from a state of persistent hyper-inflammation characterized by harmful increases in inflammatory leukocytes including macrophages. However, such studies have focused on wounds at later time points (day 10 or older), and very little attention has been given to the dynamics of macrophage responses in diabetic wounds early after injury [2], [3]. One recent report suggests that insufficient macrophage response and/or activity early after injury may also contribute to healing impairment in diabetic wound [11]. Maruyama et al [11] reported that monocyte/macrophage recruitment into the peritoneal cavity in response to thioglycolate was significantly diminished in diabetic mice compared to the control group. Although, they did not assess the macrophage responses in normal and diabetic wounds, they did demonstrate that addition of IL-1β-treated activated macrophages to diabetic wounds enhanced healing [11].

Given the importance of macrophages for the process of healing [7]–[11] and their potential role in pathology associated with impaired healing in diabetic chronic ulcers, we studied the dynamics of macrophage responses in diabetic wounds during early and late phases of healing. Our data demonstrate that, rather than a hyper-inflammatory state; the early diabetic wound exhibits a paradoxical and damaging delay in essential macrophage response. The delay in macrophage response is primarily due to insufficient chemokine expression. Importantly, one-time treatment with the pro-inflammatory chemokine CCL2 (a.k.a MCP-1), jumpstarted the macrophage response and significantly stimulated healing, suggesting that CCL2 chemokine-based therapy may be an attractive strategy to promote healing in diabetic wound.

## Results

### Macrophage response is delayed in diabetic skin tissue

We used the established and extensively used animal model for type II diabetes, C57BLKS-m *lepr^db^* (db/db) [12]–[14] and their wild-type littermates C57BL/6 (C57) to study the dynamics of macrophage response in diabetic and normal wounds respectively. In db/db mice, the gene encoding the leptin receptor (ObR) is mutated [14]. Similar mutations in leptin and the leptin receptor also occur in humans and have been linked to type II diabetes [15]. Full-thickness incisional wounds were generated on the back skin of db/db diabetic and C57 wild-type mice. Consistent with previous reports [12], [14], db/db mice developed obesity and severe diabetes with marked hyperglycemia, and exhibited impaired wound healing resembling adult-onset diabetes mellitus (Fig. S1).

**Figure 1:**
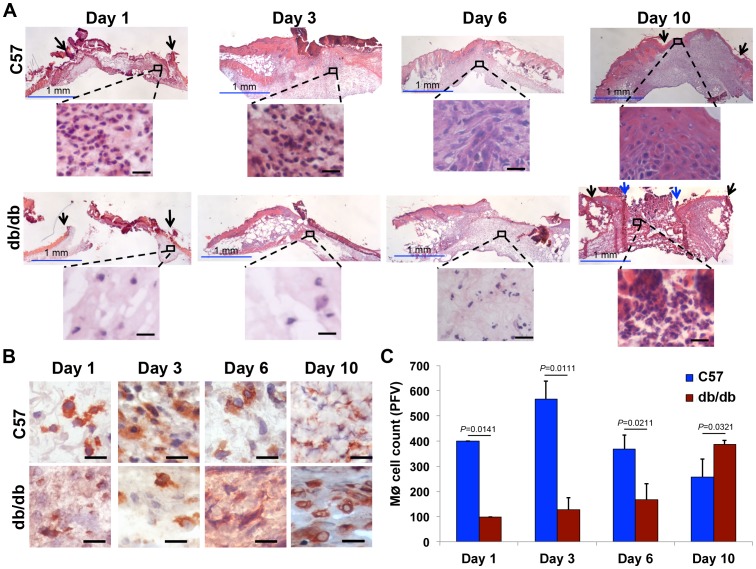
Macrophage response is delayed in diabetic skin tissue. Skin tissues from normal (C57) and diabetic (db/db) wounds were harvested at indicated time-points post wounding (day 0), fixed and stained with hematoxylin and eosin (**A**) or with CD68 antibody which primarily stains macrophages (**B**). The corresponding tabulated data for macrophage cell counts are shown as the mean ± SEM in (**C**) (N = 3 mice for H&E staining in **A**; n = 18 for **B-C.** 3 mice per group, 6 random fields per mouse from underneath the wound extending into the provisional matrix in the dermis region, all *p*-values were significant ranging from 0.0111 to 0.0321). Black arrows point to the original sites of incision. Blue arrows point to leading edges of diabetic wounds on day 10. For clarity and to enhance magnification, representative cropped regions from underneath the wounds extending in the dermis are shown. Combined, the data indicate that inflammatory response is delayed in diabetic tissues. Blue scale bar  =  1 mm, black scale bar  =  25 μm.

We harvested wound tissues from C57 and db/db on days 1, 3, 6, and 10 after wounding and carried out histological analyses using hematoxylin and eosin (H&E) tissue staining, which is frequently used to evaluate the inflammatory response in various normal and pathologic conditions including wounds [16]. Surprisingly, the results indicated that the inflammatory response was drastically delayed in db/db wounds. In line with previous reports [1], normal wounds from C57 mice exhibited infiltration of inflammatory cells on days 1 and 3 but inflammation began to subside by day 6 and was mostly resolved by day 10 (Fig. 1A). Day 10 normal wounds were fully re-epithelialized, exhibited contraction, epidermal thickening, and substantial granulation. In contrast, inflammation was minimal on days 1, 3, and 6 in db/db wounds but by day 10 post injury and consistent with previous reports [17], [18], we found diabetic wounds to contain substantially more inflammatory leukocytes than day 10 normal wounds (Fig. 1A).

To assess the dynamics of macrophage response during wound healing, we stained C57 and db/db wound tissues from days 1, 3, 6, and 10 with anti-CD68 antibody, which primarily stains macrophages [19]. Consistent with H&E staining results (Fig. 1A), we observed significant diminution in the macrophages early after injury in days 1, 3, and 6 of db/db wounds but recovery by day 10 (Figs. 1B and C, *p*≤0.0321, n = 18). CD68 antibody also reacts with myeloid precursors, peripheral blood granulocytes, and plasmacytoid DCs [19], [20]. To focus specifically on macrophages, we extracted total cells from equal amounts of C57 and db/db day 1 tissues (∼1 mm from the wound edge) and analyzed them for their macrophage cell contents (defined as CD45^+^ F4/80^+^ I-A/I-E^+^) by flow cytometry, as described [21], [22]. The data demonstrated an over 7-fold reduction in the percentage of macrophages among all leukocytes in day 1 diabetic wounds, indicating that the macrophage response, among all other leukocytes that constitute the inflammatory response, was particularly impaired in diabetic wounds early after injury (Fig. S2, *p*<0.0001, n = 5).

### Chemokine insufficiency may underlie impaired macrophage response in diabetic wounds early after injury

**Figure 2:**
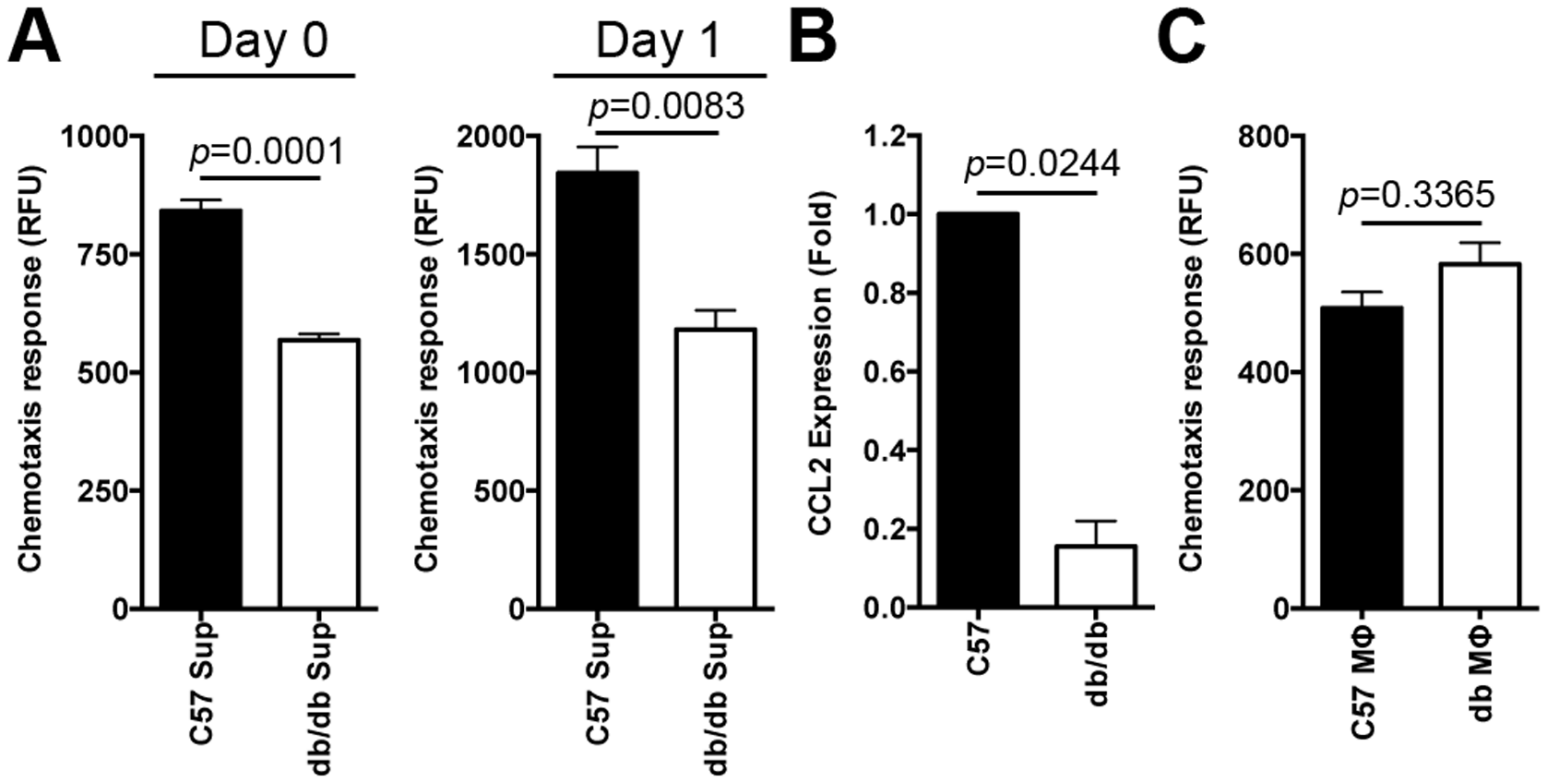
Chemokine insufficiency leads to impaired macrophage response in diabetic wounds early after injury. (**A**) Skin tissues from the normal and diabetic wound edges (1 mm from the rim) were harvested on day 0 (right after wounding) and on day 1 post wounding. These tissue explants were cultured and allowed to secrete chemokines into culture media. The supernatants from these cultures were then evaluated for their chemokine levels by assessing their ability to chemoattract normal macrophages from C57. The results are shown as the mean ± SEM (n = 18, 6 mice per group, each done in duplicates), indicating that diabetic supernatants contain significantly reduced chemoattractants for macrophages (MΦs). (**B**) CCL2 chemokine expression levels in day 1 C57 and db/db wounds were determined by RT-PCR and the results are shown as the mean ± SEM, indicating significant reduction in day 1 diabetic wounds (n = 3 mice per group). (**C**) Diabetic and normal MΦs were evaluated for their ability to chemotax toward CCL2, a known chemoattractant for MΦs. The results are shown as the mean ± SEM (n = 6, *p* = 0.3365), indicating that there is no significant differences in chemotactic responses between normal and diabetic MΦs toward CCL2.

A variety of chemokines and cytokines are expressed at wound sites and function as chemoattractants for leukocytes, although not all appear to serve indispensible physiological roles during wound healing [23]–[25]. We wished to examine whether reduced chemokine expression could account for the delayed macrophage response in db/db wounds early after injury. We utilized a functional assay to evaluate the capacity of normal and diabetic skin tissues to express chemoattractants for macrophages. Equal amounts of normal and diabetic skin tissues (∼1 mm from the edge) were harvested on day 0 (right after wounding) and on day 1 post wounding. These tissue explants were cultured and allowed to secrete chemokines into culture media as described in the Methods. The supernatants from these tissue explant cultures were then evaluated for their chemokine contents by assessing their ability to chemoattract normal macrophages from C57 mice. The results indicated that supernatants from day 0 and day 1 of diabetic skin tissues contained significantly reduced chemoattractants for macrophages. (Fig. 2A, *p*<0.0001 for day 0 and *p*<0.0083 for day 1, n = 18, 6 mice per group, each done in triplicates). CCL2 is a potent chemoattractant, which is expressed early after injury in wounds and is essential for monocyte/macrophage response and proper healing [25]–[27]. CCL2^−/−^ knockout animals exhibit significant wound healing impairment, indicating that CCL2 plays an important and non-redundant role in wound healing [25], [26]. As indicated in Figure 2B, CCL2 expression, as assessed by RT-PCR, was significantly diminished in day 1 db/db wounds (*p* = 0.0244, n = 3 mice/group).

Diabetic macrophages could also be impaired in their ability to respond to chemokines. To test this possibility, we evaluated diabetic and normal macrophages for their ability to chemotax toward CCL2, as described in the Methods. The results indicated that there were no significant differences in chemotactic responses between normal and diabetic macrophages toward CCL2 (Fig. 2C, *p* = 0.3365). Collectively, data indicated that reduction in CCL2 expression may significantly contribute to impaired macrophage response in diabetic wounds early after injury.

### CCL2 addition jumpstarts immune cell infiltration into wound and promotes healing in diabetic wounds

**Figure 3:**
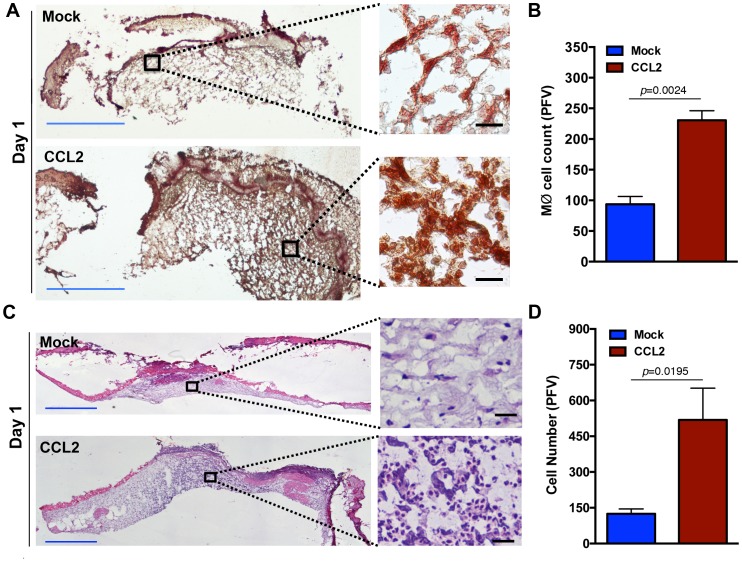
CCL2 treatment enhances monocytes infiltration in diabetic wound. Diabetic wounds were treated with PBS (Mock) or PBS + CCL2 (CCL2). Treated wound tissues were harvested on day 1 after treatment and stained with a macrophage/monocyte-specific antibody (CD68) in (**A**) or H&E in (**C**). The corresponding tabulated data for (**A**) and (**C**) are shown in (**B**) and (**D**) as mean ± SEM (N = 18, 3 mice, 6 random fields from the dermal region, *p* = 0.0024 for B and *p* = 0.0195 for D). For clarity and to enhance magnification, representative cropped regions from underneath the wounds extending in the dermis are shown. Blue scale bar  =  1 mm, black scale bar  =  25 μm.

Since insufficient CCL2 chemokine expression appeared to be the primary culprit responsible for the delayed macrophage response in diabetic wounds, we sought to restore macrophage influx in diabetic wounds by augmenting wounds with CCL2. db/db wounds were treated once at the time of injury with 60 ng CCL2, a level that is reported to be CCL2’s physiological level in normal skin wounds early after injury in mice [24], [25]. CCL2 treatment significantly enhanced macrophage infiltration in day 1 diabetic wounds (Figs. 3A and B, *p* = 0.0024, n = 18). Restoration of the macrophage response by CCL2 treatment was further corroborated by H&E histological analysis, which demonstrated substantial increases in leukocytes infiltration into the CCL2-treated diabetic wounds compared to mock-treated diabetic wounds (Fig. 3C and D, *p* = 0.0195, n = 6).

**Figure 4:**
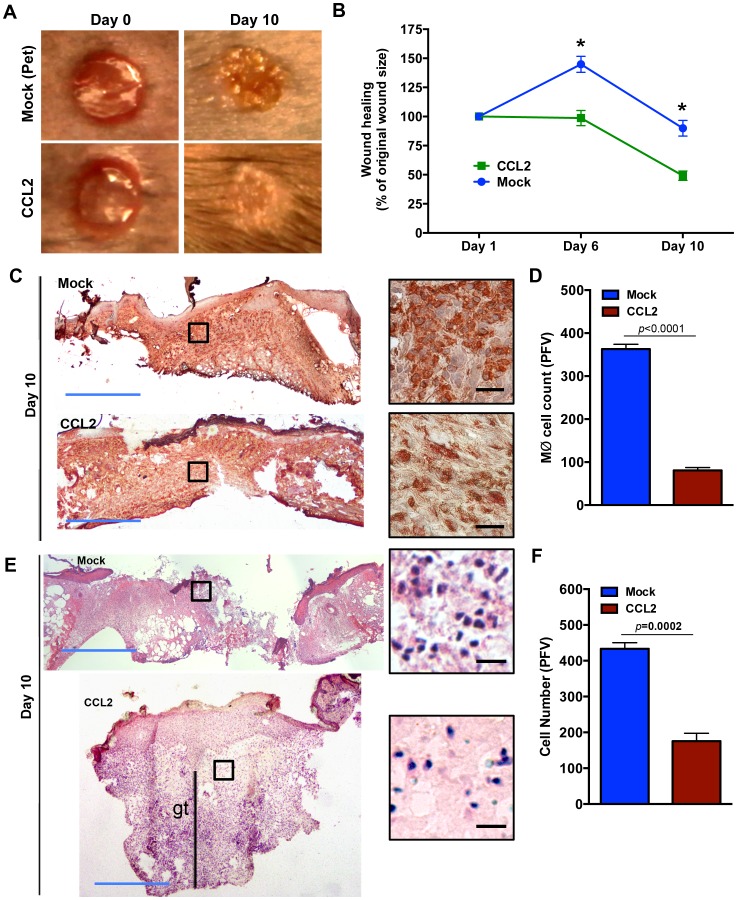
CCL2 treatment stimulates healing in diabetic wound. (**A**) Wound healing in the mock and CCL2-treated diabetic wounds was monitored by digital photography and wound areas were determined by tracing. Representative images from days 0 and 10 are shown in (**A**) and the tabulated results are shown as mean ± SEM in (**B**) (* signifies significance with *p*<0.0001, n = 4). Skin tissues from mock- or CCL2-treated diabetic wounds were harvested on day 10 after treatment, stained with monocyte/macrophage antibody (CD68) (**C**) or H&E (**E**). The corresponding tabulated data for (**C**) and (**E**) are shown in (**D**) and (**F**) as mean ± SEM (N = 18, 3 mice, 6 random fields from the dermal region, *p*<0.0001 for **D** and *p* = 0.0002 for **F**). As indicated, day 10 CCL2-treated diabetic wounds exhibit enhanced healing, complete re-epithelization, epidermal thickening, increased granulation tissue (as indicated by gt), and reduced inflammation compared to mock-treated diabetic wounds which are partially re-epithelized and are highly inflamed. For clarity and to enhance magnification, representative cropped regions from underneath the wounds extending in the dermis are shown. Blue scale bar  =  1 mm, black scale bar  =  25 μm.

Importantly, CCL2 treatment enhanced wound healing in diabetic wounds, as assessed by digital photography and wound area measurements (Figs. 4A and B, *p*<0.0001, n = 4). These results were further corroborated by histological analyses which indicated that in contrast to the mock-treated diabetic wounds that were only partially re-epithelialized by day 10, the CCL2-treated diabetic wounds were completely re-epithelialized, exhibited epidermal contraction and thickening with pronounced rete ridges, and enhanced granulation tissue (Fig 4E). Interestingly, inflammatory and macrophage responses were significantly reduced in the CCL2-treated day 10 wounds, as compared to the mock-treated wounds (Figs. 4C-4F, *p* ≤ 0.0001, n = 4).

## Discussion

Diabetic ulcers are locked in a persistent non-resolving inflammatory state, characterized by harmful increases in inflammatory leukocytes, including macrophages [2]. In this report, we provide compelling evidence demonstrating that rather than a persistent and hyper-inflammatory state that dominates diabetic chronic ulcers [17], [18], the early diabetic wound exhibits a paradoxical and damaging decrease in essential macrophage response (Fig. 1). The delay in the macrophage response in diabetic wounds results from reduced CCL2 chemokine expression (Fig. 2). Our data provide an explanation for recent findings linking impaired macrophage response and/or activity to various co-morbidities associated with diabetes. For example, it was reported that diabetic liver is more susceptible to *Listeria monocytogenes* infection [28]. This phenomenon was correlated with reduced CCL2 (MCP-1) expression in db/db liver, which suggests that inadequate macrophage response, due to CCL2 insufficiency, may be to blame for enhanced susceptibility to infection in that environment. In another study, Maruyama et al, reported reduced macrophage cell numbers in thioglycollate-induced inflammatory cells collected from the peritoneal cavity in db/db mice [11], indicating reduced macrophage infiltration in that environment. In yet another study, it was reported that granulocyte-macrophage colony-stimulating factor (GM-CSF) enhanced wound healing in diabetes by upregulating pro-inflammatory cytokines [29]. Interestingly, CCL2 was one of the upregulated pro-inflammatory cytokines.

Prolonged use of anti-inflammatory agents, has demonstrated deleterious impact on various aspects of wound healing, including an anti-proliferative effect, weakened breaking strength, reduced wound contraction, delayed re-epithelialization, impaired angiogenesis, and reduced growth factor expression [30]–[33]. Our data suggest that an opposite approach, one that is based on pro-inflammatory chemokines, may be more appropriate to stimulate healing in diabetic wounds. We find that CCL2 treatment significantly enhanced healing in diabetic wounds (Fig. 4) by restoring the macrophage response (Fig. 3). These intriguing data demonstrate that treatment with a pro-inflammatory cytokine in the early stages can have a dramatic yet positive impact on healing. Interestingly, CCL2-treated wounds did not remain inflamed and were able to resolve the inflammatory response by day 10 (Figs. 4C–4F), indicating that diabetic tissues are not destined to become locked in the persistent and hyper-inflammatory mode, which is considered an important factor contributing to impaired healing in diabetic chronic ulcer [2], [34]. These data suggest that the restoration of the proper kinetics of macrophage response may be able to jumpstart subsequent healing stages. Thus, CCL2 chemokine-based therapy may be an attractive strategy to promote healing in diabetic wound.

## Experimental Procedures

### Animal studies procedures

All procedures complied strictly with the standards for care and use of animal subjects as stated in the Guide for the Care and Use of Laboratory Animals (Institute of Laboratory Animal Resources, National Academy of Sciences, Bethesda, MD, USA). We have an approval from the Rush University Medical Center Institutional Animal Care and Use Committee (IACUC No: 10-094) to conduct research as indicated. A sterile biopsy punch (3- or 5-mm diameter, Acuderm**®** Inc. Lauterdale, FL) was used to punch through the full thickness of the back skin below the shoulder blades in normal (C57Bl/6) mice and their diabetic littermates, C57BLKS-m *lepr^db^* (*db/db*). For wound healing assessment, transparency paper was used to trace the wound edges. The wound areas were subsequently calculated using NIH ImageJ software [35]. Wound healing was assessed at 24 hr interval by digital photography and expressed as % wound remaining, calculated as the percentage of original wound area {(open wound area/initial wound area) X 100}. For immunohistological (IHC) studies, wound tissues were harvested, snap-frozen and embedded in Tissue-Tek O.C.T. (Sakura), or fixed in 10% formalin, transversely cut into 7-μm-thick sections from the middle part of the wounds, stained with hematoxylin and eosin (H&E) or CD68 antibody which primarily stains macrophages.

### RNA extraction from tissues

Skin tissues were placed into a Lysing Matrix D tube from MP Biomedicals (116913100) along with 1 ml of Trizol from Invitrogen. Tissue was disrupted using MP Biomedicals FastPrep Automated Homogenizer, 10 cycles at 6.0 m/s for 40sec. The protocol for Trizol was then followed to isolate RNA. RNA was column purified, using BioRad Total RNA Mini Kit.

### Tissue digestion and flow cytometric analysis

C57 and db/db skin tissues were obtained on day 1 via 5 mm punch biopsies, weighed, and place immediately in cold HBSS (Mediatech, Inc., Manassas, VA). Subcutaneous fat was removed using a scalpel and scissors were used to cut the tissue into small (<2 mm) pieces. The tissue was enzymatically dissociated in DNAse I (40 μg/ml; Sigma-Aldrich Co., St. Louis, MO) and Colagenase D (1 mg/ml HBSS; Roche Diagnostics, Indianapolis, IN) at 37°C for 30 minutes. Cold PBS was used to stop the dissociation process. The tissue was then mechanically dissociated using the gentleMACS octoDissociator (Program B; Miletynyi Biotec, Auburn, CA) and passed through 70 μm nylon screens into 50 ml conical tubes. Cells were then washed twice with PBS. Resultant single-cell suspensions were stained using fluorescently labeled antibodies against cell surface markers, according to standard protocols described previously [22], [36]. All antibodies were purchased from eBioscience, Inc. (San Diego, CA). Flow cytometry was performed using a BD FACS Canto II flow cytometer (BD Biosciences, San Jose, CA) and data were analyzed using FlowJo software (Tree Star, Ashland, OR). For the gating strategy, live leukocytes were identified by using a lymphocyte (FSC-A vs SSC-A) gate followed by a CD45 versus Live/Dead gate (where leukocytes are CD45^+^ and live cells are negative for Live/Dead). Doublets were then excluded using SSC-A vs. SSC-H and FSC-A vs. FSC-H gating. Lastly, neutrophils were identified as Ly6C/G^hi^CD11b^hi^ cells and macrophages were identified as non-neutrophil F4/80^+^I-A/I-E^+^ cells [37], [38].

### Functional analyses of tissue chemokine content

The ability of normal and diabetic wound tissues to express chemoattractants was assessed using a technique described previously [39]. Briefly, equal amounts of day 1 wound tissues from C57 and db/db were harvested and incubated in 5 ml of Keratinocyte serum-free defined media (Invitrogen) for 4 days to allow expression and secretion of chemokines into the culture. The chemokine activities contained within the aforementioned culture supernatants (Sup) were then tested for their ability to attract normal or diabetic macrophages in a chemotaxis assay. We used a previously described method [40] to isolate murine macrophages. Briefly, bone-marrow from the femurs of C57 or db/db mice were grown on low-attachment plates for 7days in complete RPMI with 20 ng/ul Macrophage- Colony Stimulating Factor (M-CSF) obtained from Miltenyi. Macrophages were labeled with calcein AM (Invitrogen) and a chemotaxis assays using wound cultured media as the chemoattractant was performed with a 96-well ChemoTx plate (NeuroProbe) for 1hr at 37°C as described previously [41]. Fluorescence of transmigrated macrophages was determined by quantifying fluorescence on a plate reader (BioTek).

### Gene expression analysis by Real-time polymerase chain reaction (RT-PCR)

Gene expression was assessed by RT- PCR: cDNA was generated using iScript™ cDNA Synthesis Kit from Bio-Rad, according to manufacturer’s protocol. RT-PCR was then preformed with gene-specific primer pairs obtained from Qiagen (QuantiTect primer assay, Cat. No: QT00167832 (CCL2), QT01658692 (GAPDH)), using the Applied Biosystems 7900HT Fast Real-Time PCR system. The data were calculated using the 2^−ΔΔCt^ method and were presented as ratio of transcripts for gene of interest normalized to GAPDH or tissue weight.

### Serum glucose level determination

Mouse tail-vein blood was used to sample blood glucose levels with a FreeStyle Lite glucose monitor (Abbott).

### Reagents

Antibodies against GAPDH from Sigma (G9545). Antibodies against Ly6G and CD68 were from Abcam. Primer pairs for GAPDH were from Qiagen. CCL2 chemokine was from ProSpec in lyophilized form and was re-suspended in PBS.

### Statistical analysis

Statistical analyses were performed by One-way analysis of variance, Bartlett's test for equal variances, or Student t-test, using the GraphPad Prism software. Data are presented as mean ± SEM. *P*-values less than or equal to 0.05 were taken as significant.

## Supporting Information

Figure S1 **Characterization of db/db diabetic animal model used in these studies.** (**A**) Normal (C57) and diabetic (db/db) animals were weighed prior to wounding. (**B**) The blood glucose levels of C57 and db/db animals were determined prior to wounding. (**C**) Wound healing db/db and C57 mice were monitored daily by digital microscopy for 10 days. Representative images from day 0 and day 10 are shown. (**D**) Wound healing was measured by ImageJ and the tabulated results are shown as the mean ± SEM (n ≥ 12 mice per group, * indicates significance with *p*≤0.0135). As indicated by this figure, diabetic animals are obese, have higher serum glucose levels, and are severely impaired in wound healing.
(TIF)

Figure S2 **Macrophage response is impaired in diabetic wound early after injury.** (**A**) The gating strategy for determining the macrophage subpopulations in skin tissue. Skin tissues were harvested from C57 or db/db day 1 wounds. Live leukocytes were identified by using a lymphocyte (FSC-A vs SSC-A) gate followed by a CD45 versus Live/Dead gate (where leukocytes are CD45+ and live cells are negative for Live/Dead). Doublets were then excluded using SSC-A vs SSC-H and FSC-A vs FSC-H gating. Lastly, neutrophils were identified as Ly6C/G^hi^CD11b^hi^ cells and macrophages were identified as non-neutrophil F4/80^+^I-A/I-E^+^ cells. Day 1 wound tissues (∼1 mm from the rim) were harvested from C57 and db/db and the percentage of macrophages among all leukocytes were determined by flow cytometry, using the gating strategy described in (**A**). Representative flow histograms are shown in (**B**) and the corresponding data are shown in as mean ± SEM. (N  =  5 mice/group, *p*<0.0001).
(TIF)
