# Supplementary figures and images for: Pro-Inflammatory Chemokine CCL2 (MCP-1) Promotes Healing in Diabetic Wounds by Restoring the Macrophage Response
Source: PLoS One. 2014 Mar 11;9(3):e91574. doi: 10.1371/journal.pone.0091574 (PMC3950222; doi:10.1371/journal.pone.0091574)

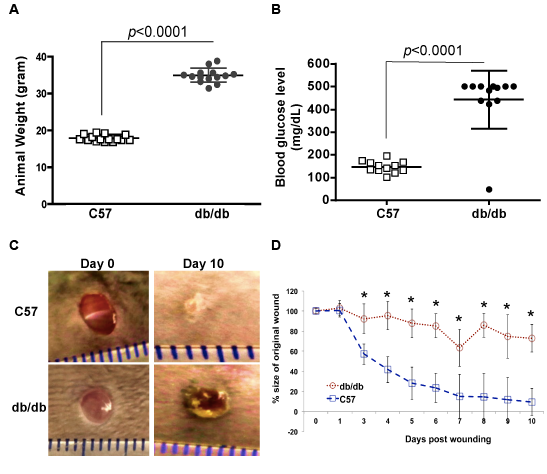

Supplement: Figure S1 — Characterization of db/db diabetic animal model used in these studies. (A) Normal (C57) and diabetic (db/db) animals were weighed prior to wounding. (B) The blood glucose levels of C57 and db/db animals were determined prior to wounding. (C) Wound healing db/db and C57 mice were monitored daily by digital microscopy for 10 days. Representative images from day 0 and day 10 are shown. (D) Wound healing was measured by ImageJ and the tabulated results are shown as the mean ± SEM (n ≥ 12 mice per group, * indicates significance with p≤0.0135). As indicated by this figure, diabetic animals are obese, have higher serum glucose levels, and are severely impaired in wound healing. (TIF) [file pone.0091574.s001.tif]

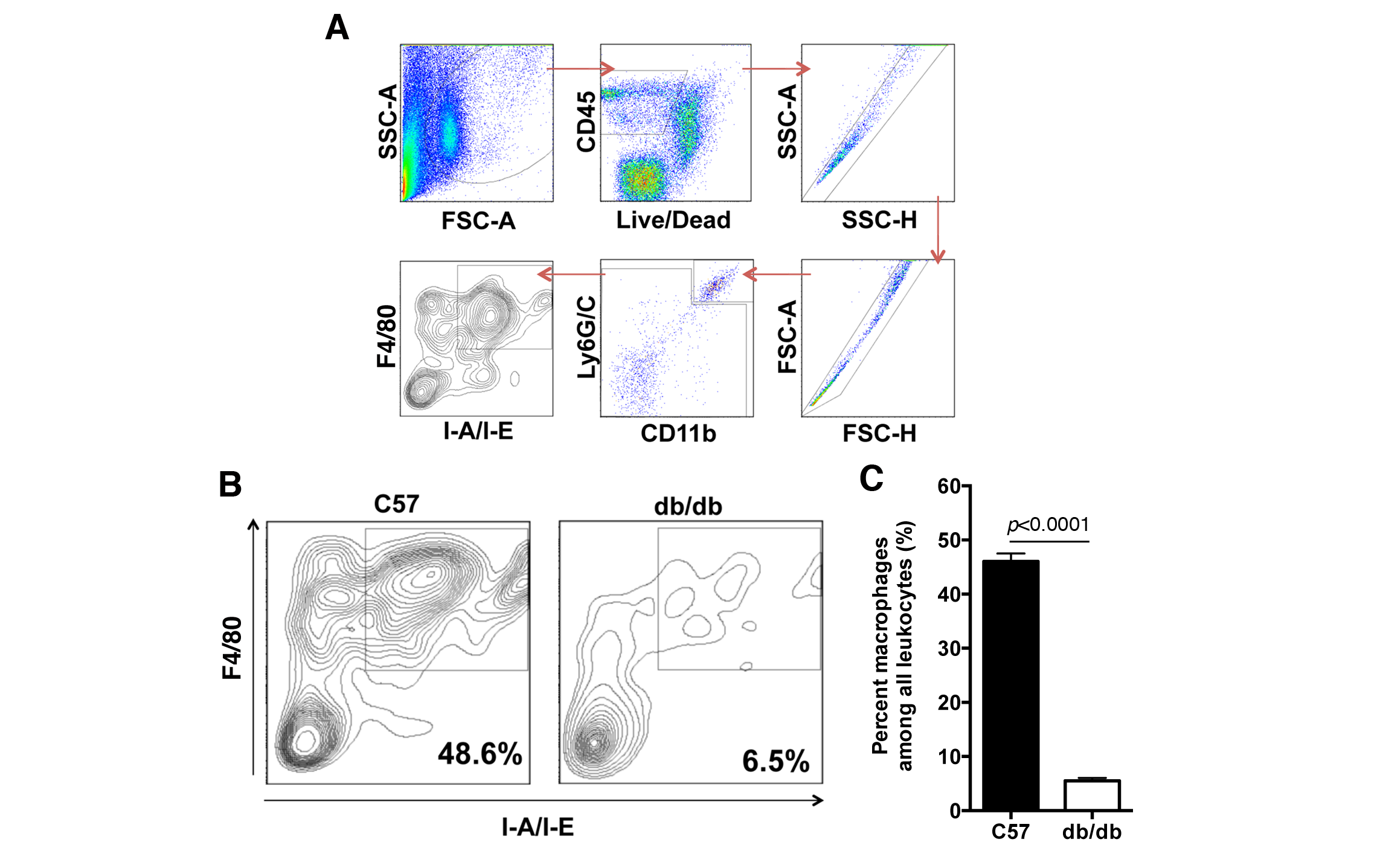

Supplement: Figure S2 — Macrophage response is impaired in diabetic wound early after injury. (A) The gating strategy for determining the macrophage subpopulations in skin tissue. Skin tissues were harvested from C57 or db/db day 1 wounds. Live leukocytes were identified by using a lymphocyte (FSC-A vs SSC-A) gate followed by a CD45 versus Live/Dead gate (where leukocytes are CD45+ and live cells are negative for Live/Dead). Doublets were then excluded using SSC-A vs SSC-H and FSC-A vs FSC-H gating. Lastly, neutrophils were identified as Ly6C/GhiCD11bhi cells and macrophages were identified as non-neutrophil F4/80+I-A/I-E+ cells. Day 1 wound tissues (∼1 mm from the rim) were harvested from C57 and db/db and the percentage of macrophages among all leukocytes were determined by flow cytometry, using the gating strategy described in (A). Representative flow histograms are shown in (B) and the corresponding data are shown in as mean ± SEM. (N = 5 mice/group, p<0.0001). (TIF) [file pone.0091574.s002.tif]
